# Combination of seed priming and nutrient foliar application improved physiological attributes, grain yield, and biofortification of rainfed wheat

**DOI:** 10.3389/fpls.2023.1287677

**Published:** 2023-10-31

**Authors:** Layegh Moradi, Adel Siosemardeh

**Affiliations:** Department of Agronomy and Plant Breeding, Faculty of Agriculture, University of Kurdistan, Sanandaj, Iran

**Keywords:** antioxidant, biofortification, grain yield, malondialdehyde, micronutrients, silicon, terminal stage

## Abstract

Seed priming and foliar application are two crop management practices that can increase grain yield and quality. The research aimed to assess the influence of seed priming and foliar application on rainfed wheat. Two field experiments with two seed priming rates (control and priming) and five foliar applications [control, urea (4%), silicon (4 mM), FeSO_4_.7H_2_O (0.6%), and ZnSO_4_.7H_2_O (0.4%)] at the anthesis/Z61 stage were conducted. Seeds were primed for 12 h at 25 ± 2°C, by soaking in an aerating solution [urea (20 g L^−1^) + FeSO_4_.7H_2_O (50 ppm) + ZnSO_4_.7H_2_O (50 ppm) + silicon (20 mg L^−1^)]. Seed weight-to-solution volume ratio was 1:5 (kg L^−1^). A pot experiment was also conducted to examine the effect of priming on root growth. Overall, combined seed priming and foliar application induced a positive impact on physiological traits and attributes. Maximum chlorophyll a, chlorophyll b, total chlorophyll, and carotenoid concentrations (1.58, 0.669, 2.24, and 0.61 mg g^−1^ FW), membrane stability index (77.31%), superoxide dismutase and peroxidase activity (0.174 and 0.375 Unit mg^−1^ protein), 1,000-grain weight (35.30 g), biological yield, grain yield (8,061 and 2,456 kg ha^−1^), and minimum malondialdehyde concentration (3.91 µg g^−1^ FW) were observed in seed priming combination with ZnSO_4_ foliar application. The highest glycine betaine concentration (6.90 mg g^−1^ DW) and proline (972.8 µg g^−1^ FW) were recorded with the co-application of seed priming and foliar urea spraying. Foliar application of ZnSO_4_, FeSO_4_, and urea drastically enhanced grain Zn (29.17%), Fe (19.51%), and protein content (increased from 11.14% in control to 12.46% in urea foliar application), respectively. Compared to control, seed priming increased root length, root volume, and dry mass root by 8.95%, 4.31%, and 9.64%, respectively. It is concluded that adequate Zn, Fe, silicon, and N supply through seed priming and foliar applications of these compounds at the terminal stage of rainfed wheat alleviates drought stress and improves GY and biofortification.

## 1 Introduction

In general, approximately 20% of the calories needed by the global community are derived from wheat (*Triticum aestivum* L.), which is one of the main sources of food in the world (Liu et al., 2020). The demand for wheat production is rising in response to a growing global population and food consumption growth (Liu et al., 2015). Water scarcity has the greatest impact on agriculture, with yields in rainfed areas decreasing by 40% to 60% (Saha et al., 2022). During the wheat-growing season, uneven precipitation distribution leads to drought stress in the Mediterranean. As a result, rainfed wheat growth is negatively impacted by soil moisture shortages, especially during anthesis and grain filling (Moradi et al., 2022). Crop producers must maintain crops’ ability to adapt to frequent stressors (Malko et al., 2022). Integrated agronomic crop management methods are needed to alleviate the deleterious impacts of drought and enhance quantitative and qualitative yields. The singleton approach will not achieve this (Melash et al., 2019). For this goal, we combined seed priming and foliar application of urea, ZnSO_4_, FeSO_4_, and silicon in an experiment. Research was conducted on the effects of these factors on physiological characteristics and wheat yield quantitatively and qualitatively.

Seed priming practice has been utilized in plants to boost the quality of seed, thereby benefiting stand establishment and mitigating drought stress in farmers’ fields (Liu et al., 2015). The technique involves partially hydrating seeds in a specified environment until the germination process begins without exhibiting any signs of radical emergence. It is followed by dehydrating to attend to the original seed’s dry weight. In addition, priming can activate metabolic systems normally activated during germination (“pre-germinative metabolism”) and therefore accelerate germination and emergence, and therefore allows seedlings to adapt to various environmental stresses. Typical priming approaches include water (Hydro) priming, osmopriming, hormonal priming, and nutritional priming (Liu et al., 2015; Rai-Kalal and Jajoo, 2021). Seeds are soaked in a solution containing nutrients during nutritional priming. Soil, leaf, and seed nutrients may be applied to fulfill plant nutrient demands (Farooq et al., 2012; Rehman et al., 2018; Farooq et al., 2021). Fertilizer applied to soil takes effect slowly (5–6 days) if environmental variables are favorable. It is difficult to apply fertilizers uniformly to the surface of the soil in a soil application (Johnson et al., 2005). It is advantageous to prime seeds so that nutrients can easily be accessed by germinating seeds, and it is also considered a cost-efficient method because very few nutrients are required to prime seeds (Farooq et al., 2021). Seed priming contributes to enhance drought resistance via increased photosynthesis pigments, antioxidant defense, osmotic adjustments, and membrane integrity (Saha et al., 2022). Research has documented that in wheat plants, seed priming boosts nutrient uptake, antioxidant enzymes, osmolyte concentrations like glycine betaine (GB) and proline, chlorophyll content, membrane integrity, yield characteristics and yield, and grain nutrient and protein concentrations (Farooq et al., 2012; Rehman et al., 2018; Reis et al., 2018; Malko et al., 2022).

Drought stress becomes more intensified under mineral nutrient deficiencies (Farooq et al., 2021). Rainfed wheat development is typically impacted by drought stress in Mediterranean regions due to unbalanced rainfall distribution (Moradi et al., 2022). In such regions, foliage application nutrients are a suitable technique to mitigate adverse drought stress effects and improve rainfed wheat physiological characteristics, grain yield (GY), and quality. Foliar nutrition is a method proposed to reduce chemical fertilizers and environmental risks (Aziz et al., 2019; Melash et al., 2019). In rainfed wheat farming, where the plant has difficulty absorbing nutrients from the soil in the final stages of its growth due to low soil moisture and reduced root activity, this method can be very effective in supplying nutrients (Melash et al., 2019).

Nitrogen (N) optimal utilization is vital for enhanced wheat grain and protein production (Nehe et al., 2020). Because of the utilization of N during the flowering and grain-filling stages, nutrients tend to be transported efficiently to higher metabolic demand points (grain) (Blandino et al., 2020). During the terminal stages of growth, N intake has less effect on yield but a significant influence on grain quality (Wang et al., 2021). Silicon enhances crop yield, quality, and productivity by improving photosynthetic activity and N assimilation. The International Plant Nutrition Institute identifies silicon as a beneficial mineral based on its useful role in plants. Owing to the potential for accumulation, plants collect silicon between 0.1% and 10% of their dry matter. It is, however, not recognized as a necessary nutrient for proper plant development and growth (Bukhari et al., 2021; Sattar et al., 2021). Research has found silicon to have a positive impact on various plant species, notably when exposed to environmental stress, by elevating antioxidant enzyme activity and osmolytes, which help plants resist abiotic and non-abiotic stresses (Bukhari et al., 2021). Zn and Fe are indispensable micronutrients for crop growth. Many scientists have documented that foliage application of these minerals is able to amplify drought tolerance of plants due to their participation in the construction of many antioxidant enzymes as cofactors, raising GY, and enriching wheat grains (Karim et al., 2012; Zain et al., 2015; Sultana et al., 2018; Melash et al., 2019; Sattar et al., 2021). Optimal fertilizer usage and the value of micronutrients for soil and human wellbeing have been carefully discussed in recent years, and with the change of approach toward nutrient management, rather than focusing exclusively on boosting yields, it also addresses its impact on society. In addition to increasing yields per hectare, it also emphasizes its impact on people, aiming to diminish malnutrition in countries and societies, and most noteworthy, it emphasizes the role of Zn and Fe (Das et al., 2022; Szerement et al., 2022). Thus, the utilization of such micronutrients in crop production increases GY and product quality, which will result in better health for humans.

Several studies have examined wheat priming and foliar applications of nutrients (Amanullah et al., 2010; Farooq et al., 2012; Zain et al., 2015; Rehman et al., 2018; Melash et al., 2019; Farooq et al., 2021). Nevertheless, there is limited information regarding the effects of simultaneous seed priming and continued foliar fertilization on physiological characteristics, yield attributes, and grain quality; such knowledge would be extremely useful for managing nutrients with the aim of reducing drought stress in rainfed wheat crops and improving yields and quality. Here, we evaluate the influence of priming and foliar spray of N, Zn, Fe, and silicon on rainfed wheat. The hypothesis was that seed priming and foliar application of nutrients could improve root growth, morpho-physiological traits, productivity, and grain quality in rainfed wheat crops. Therefore, the objectives of our experiment are to (a) assess the effect of priming on root attributes; (b) assess the effect of priming, foliar application of nutrients, and their interaction under rainfed conditions on the photosynthetic pigments, compatible osmolytes, malondialdehyde (MDA), superoxide dismutase (SOD), and peroxidase (POD) activity; (c) investigate the effect of foliar application nutrients and priming on the rainfed wheat yield components and yield; (d) compare grain Fe, Zn, and protein content (grain quality) of wheat grain under the influence of priming and foliar minerals spraying; and (e) examine relationships between physiological traits, yield, and yield attributes.

## 2 Materials and methods

### 2.1 Field experiment

#### 2.1.1 Site descriptions

The field experiment was conducted at the University of Kurdistan’s research farm, which is situated on the Dehgolan plain (35 −19′ 1′′ N, 47 −18′ 54′′ E, 1,862 m above mean sea level) during the wheat-growing seasons of 2021–2023. **Table 1** shows organic C, available phosphorus, total N, and available potassium, Zn, and Fe in the 0–0.3 m soil layer of the field. The research field soil texture was loam. During the growing seasons of 2021–2022 and 2022–2023, 262.4 and 368.6 mm of precipitation were recorded. In **Table 2**, the monthly precipitation, temperature, and average relative humidity at the wheat-growing site are presented.

**Table 1:** Pre-sow nutrient condition of experimental plots at 0–0.30 m depth.

Growing season	Organic C (%)	Total N (g kg^−1^)	Available P (mg kg^−1^)	Available K (mg kg^−1^)	Zn (mg kg^−1^)	Fe (mg kg^−1^)
**2021–2022**	0.78	0.40	12.00	336.1	0.44	2.10
**2022–2023**	0.81	0.45	12.80	351.0	0.42	2.03
**Pot experiment**						
**2022–2023**	0.73	0.37	13.10	330.0	0.38	2.30

**Table 2:** Wheat-growing season rainfall, minimum and maximum temperatures (T_min_; T_max_), and mean relative humidity (RH_avg_).

Month	2021–2022				2022–2023			
	Precipitation (mm)	T_min_	T_max_	RH_avg_ (%)	Precipitation (mm)	T_min_	T_max_	RH_avg_ (%)
		(°C)				(°C)		
**Oct.**	21.4	1.77	21.16	44.20	5.60	3.70	23.57	41.63
**Nov.**	32.4	−1.11	10.63	72.79	36.4	−2.03	12.85	63.36
**Dec.**	24.2	−4.32	9.00	65.39	22.00	−3.80	4.80	80.03
**Jan.**	32.4	−9.76	4.6	68.61	50.40	−11.06	0.18	83.50
**Feb.**	74.6	−6.30	6.79	74.16	15.20	−10.33	3.68	72.90
**Mar.**	34.6	−2.03	10.86	52.39	106.00	0.80	13.42	56.94
**Apr.**	33.8	2.68	19.77	45.89	81.20	1.75	17.36	54.06
**May.**	8.6	4.09	21.29	49.75	76.60	5.38	21.15	51.25
**Jun.**	0.4	8.25	30.79	35.24	5.2	7.14	28.49	39.87
**Total**	262.4	–	–	–	398.60	–	–	–

#### 2.1.2 Experimental design

In the two growing seasons, this study was conducted in factorial with four replications utilizing a randomized complete block design (RCBD) to assess the effect of seed priming and foliar application on rainfed wheat (Cv. Baran). The experimental factors included two priming levels (control and priming with) and five foliar applications [control, urea (4%), silicon (4 mM), FeSO_4_.7H_2_O (0.6%), and ZnSO_4_.7H_2_O (0.4%)]. Each plot was 2.9×8 m^2^, and the distance between plots and blocks was 1 m and 2 m, respectively.

Seeds were primed for 12 h at 25 ± 2°C, by soaking in an aerating solution [urea (20 g L^−1^) + FeSO_4_.7H_2_O (50 ppm) + ZnSO_4_.7H_2_O (50 ppm) + silicon (20 mg L^−1^)]. The ratio of seed weight to solution volume was 1:5 (kg L^−1^). During the soak, an aquarium pump supplied aeration. To ensure clump-free sowing of the seeds, the seeds were dried in the shade after being removed from the solution. Two foliar applications were performed, first at the anthesis/Z61 (Zadoks et al., 1974) stage and again 10 days later, using a manual high-pressure sprayer at a rate of 500 L ha^−1^. The control plots were sprayed with water. A water spray was applied to the control plots.

#### 2.1.3 Crop management

During seeding, plots were fertilized with 150 kg ha^−1^ of urea and 200 kg ha^−1^ of triple superphosphate. Wheat seeds were sown at a density of 350 plants per m^2^. Tilling, planting, pest control, and weed control are similar to those used in typical wheat production.

#### 2.1.4 Measurements

Physiological characteristics were measured 8 days after the last foliar application. To determine parameters such as chlorophyll pigment, proline, GB, MDA concentration, SOD and POD activity, flag leaf samples were randomly collected in each plot. Liquid nitrogen was used to freeze samples and they were stored at −40°C until analysis. We measured the membrane stability index (MSI) after separating flag leaves from plots to study this parameter.

##### 2.1.4.1 Carotenoids and chlorophyll content

A method based on Arnon’s (1949) method was used to determine chlorophyll in flag leaves. We homogenized the leaf samples in 10 mL of 80% acetone and centrifuged them at 5,000 rpm for 5 min. The extract absorbance was recorded at 645-nm (A645), 663-nm (A663), and 470-nm (A470) wavelengths using a spectrophotometer (UV-2100 Model).

##### 2.1.4.2 Proline concentration

Based on Bates et al. (1973), proline concentration was measured. In 10 mL of 3% (w/v) sulfosalicylic acid solution, samples (0.5 g) were homogenized. For 1 h at 100°C, 2 mL of the extract reacted with 2 mL of glacial acetic acid and 2 mL of fresh acid ninhydrin solution in a test tube, and the reaction was finished in an ice bath. A spectrophotometer (UV-2100 Model) was used to measure the supernatant absorbance at 520 nm after adding 4 mL of toluene.

##### 2.1.4.3 GB concentration

The GB was extracted from dry flag leaves using hot distilled water (70°C). Dry flag leaves were soaked in distilled water for 48 h and then shaken vigorously to measure GB. 2N HCl and potassium tri-iodide solution were added to the extract (0.25 ml). A 90-min ice bath was used to cool the contents after shaking. Following this, 20 mL of 1,2-dichloromethane (cooled at −10°C) and 2 mL of ice-cold distilled water were added. There were two layers formed from the mixture; 365-nm optical density measurements were performed after discarding the upper aqueous layer (Grieve and Grattan, 1983).

##### 2.1.4.4 MDA concentration

The Heath and Packer (1968) method was used to determine MDA content in leaf. Five milliliters of 0.1% trichloroacetic acid (TCA) was homogenized with leaf samples (0.5 g), followed by centrifugation at 10,000 *g* for 5 min at 4°C.

##### 2.1.4.5 SOD activity

Superoxide dismutase activity was determined based on Beauchamp and Fridovich (1971). Each sample was prepared using 970 μL of mixed buffer, 20 μL of riboflavin, and 20 μL of extract. For 10 min, the samples were shaken under the light. Using a spectrophotometer, the absorbance at 562 nm was measured at the end.

##### 2.1.4.6 POD activity

Peroxidase activity was determined based on Hemeda and Klein (1990). In an overall volume of 1.0 mL, the reaction mixture contained 780 µL of 50 mmol potassium phosphate buffer (pH 6.6), 90 µL of 0.3% hydrogen peroxide, 40 µL of crude extract, and 90 µL of 1% guaiacol. The activity was detected by the rise in absorption at 470 nm in response to guaiacol oxidation (E026.6 mM^−1^ cm^−1^).

##### 2.1.4.7 MSI

To determine the MSI, leaf discs were cut with scissors and inserted into glass vials. The samples were washed twice with double-distilled water for 5 min each. Following draining the water from each vial, 10 mL of double-distilled water was added. The vials were then shaken (150 rpm, 25°C, 30 min). The electrical conductivity was measured using a conductivity electrode. The conductivity was measured after 60 min of hot water bathing all vials. According to Bajji et al. (2002), the MSI was calculated.

##### 2.1.4.8 Yield components and yields

Grain yield at maturity was determined by cutting 3 m^2^ of each plot in the center and expressing it at 12% moisture content. Divide GY by biological yield (BY) at maturity to calculate the harvest index (HI). We also measured the kernels per spike at maturity and the 1,000-grain weight.

##### 2.1.4.9 Fe and Zn content

The dried wheat grain samples were ashed at 550°C in a furnace for 6 h, then dissolved in a 1:1 (v:v) HNO_3_ solution to determine Fe and Zn content. Atomic absorption spectrometry was used to detect Fe and Zn in grains (Jones and Case, 1990).

##### 2.1.4.10 Grain protein content

To measure grain protein content, the N concentration was initially measured with the Kjeldahl method. By multiplying the grain N content by the N-to-protein conversion factor (5.7), the grain protein content was calculated (Moradi et al., 2022).

#### 2.1.5 Statistical analysis

In order to analyze the combined variance of the data, after Bartlett’s test and ensuring homogeneity of variance, the factorial combined analysis model based on the RCBD was used. Years were considered random, and seed priming and foliar application were used as fixed effects. Using SAS 9.4 software, we conducted an analysis of variance. To compare the means, Duncan’s multiple range test was used at a *p* ≤ 0.05 (Duncan, 1966). To assess the relationship between the variables, Pearson correlation coefficients were employed.

### 2.2 Pot experiment

#### 2.2.1 Plant materials and experiment setup

The pot experiment was carried out during the 2022–2023 growing season at the University of Kurdistan, Kurdistan, Iran. The purpose of this experiment was to evaluate the effect of seed priming on root growth in rainfed wheat (Cv. Baran). The pot experiment was conducted with soil obtained from the field. **Table 1** shows some of the physical and chemical characteristics of the medium soil. The experiment was designed as a 4-replicate completely randomized design with seed priming treatment [non-primed and primed seeds with urea (20 g L^−1^) + FeSO_4_.7H_2_O (50 ppm) + ZnSO_4_.7H_2_O (50 ppm) + silicon (20 mg L^−1^)]. As described in *section 2.1.2*, the priming of seeds was done the same way in the field experiment. First, plastic pots (12 in diameter and 100 cm in height) were filled with 15 kg of soil (**Table 1**). A pot was placed in the excavated ground and the surrounding area was filled with soil so that it had the same surface as a field. Three seedlings remained per pot after seedling establishment, and they were grown to physiological maturity. Plants did not show any signs of disease or pest activity.

#### 2.2.2 Plant growth attributes

At maturity, plant dry mass (DM) and grain weight were measured in grams for each pot in this experiment. The pots were removed from the ground after harvesting the aboveground part at maturity in order to measure root length, root volume, and root dry weight. For 12 h, the pots were placed in water-filled barrels. Roots were stirred and poured into a sieve (0.25 mm^2^ mesh). As the sieve was suspended over a water bath, it was repeatedly shaken until soil was removed from the roots. The remaining soil materials were manually removed from the sieve. The measured root length, root volume, and root DM were then averaged. Root length was calculated using a ruler from the base to the tip, and total measurements were taken. Root masses were placed in a water-filled measuring cylinder to determine root volume. Water level increase was measured as cm^3^ pot^−1^. For each pot, roots were oven dried for 48 h at 72°C, and root DM was calculated as g pot^-1^.

#### 2.2.3 Statistical analysis

Statistical Analysis Software (SAS Version 9.4) was used to analyze the data using a completely randomized design (ANOVA). To compare the means, Duncan’s multiple range test was used at a *p* ≤ 0.05 (Duncan, 1966).

## 3 Results

### 3.1 Variance analysis of studied characteristics

A combined ANOVA for traits revealed that there was a significant year, seed priming, and foliar application effect for almost all traits (**Table 3**). Moreover, the interactions between seed priming × foliar application for Chl a, Chl b, Chl T, and carotenoid content, proline, GB, and MDA concentration, POD and SOD activity, 1,000-grain weight, BY, and GY were also significant (**Table 3**).

**Table 3:** Analysis of variance (ANOVA) of factorial design for wheat studied wheat traits at two priming rates and foliar applications.

			Year (Y)	Priming (P)	Foliar application (FA)		Y×P	Y×FA		P × FA		Y × P × FA
Degrees of freedom (df)			1	1	4		4	4		4		4
**Physiological and biochemical**	Chlorophyll a		**	**	**		ns	ns		*		ns
	Chlorophyll b		**	**	**		ns	ns		*		ns
	Total chlorophyll		**	**	**		ns	ns		**		ns
	Carotenoid		**	**	**		ns	ns		**		ns
	Membrane stability index		**	*	**		ns	ns		**		ns
	proline		*	**	**		ns	ns		**		ns
	Glycine betaine		*	**	**		ns	ns		*		ns
	Malondialdehyde		**	**	**		ns	ns		**		ns
	Peroxidase activity		**	**	**		ns	ns		*		ns
	Superoxide dismutase		ns	**	**		ns	ns		**		ns
	Spike per m^2^		**	**	ns		ns	ns		ns		ns
**Yield components and yield**	Kernels per spike		**	**	**		ns	ns		ns		ns
	1,000-grain weight		**	**	**		ns	ns		**		ns
	Biological yield		**	**	**		ns	ns		*		ns
	Grain yield		**	**	**		ns	ns		*		ns
	Harvest index		ns	**	**		ns	ns		ns		ns
**Grain quality**	Protein		ns	*	**		ns	ns		ns		ns
	Zn		ns	**	**		ns	ns		ns		ns
	Fe		ns	**	**		ns	ns		ns		ns
**Mean comparisons**	Chl a	Chl b	Chl T	Carotenoid	Proline (ug g^−1^ FW)	GB (mg g^−1^ DW)	MSI (%)		MDA (µg g^−1^ FW)		POD (Unit mg^−1^ protein)	
	(mg g FW^−1^)											
**Year**												
**2021–2022**	1.30^b^ ± 0.02	0.50^b^ ± 0.010	1.79^b^ ± 0.027	0.48^b^ ± 0.018	818^a^ ± 14.3	6.08^a^ ± 0.10	68.5^b^ ± 0.68		5.17^a^ ± 0.11		0.318^a^ ± 0.006	
**2022–2023**	1.48^a^ ± 0.02	0.59^a^ ± 0.012	2.06^a^ ± 0.031	0.55^a^ ± 0.010	767^b^ ± 21.1	5.86^b^ ± 0.13	72.7^a^ ± 0.93		4.80^b^ ± 0.10		0.307^b^ ± 0.007	

ns, non-significant error within-group variance. *: p ≤ 0.05; **: p ≤ 0.01. Mean comparisons for chlorophyll a, chlorophyll b, total chlorophyll, and carotenoid concentration, membrane stability index, proline, glycine betaine, malondialdehyde, and peroxidase activity. As determined by Duncan’s test, no significant difference at p ≤ 0.05 exists between values in a column containing the same letter within a group. Data are the mean ± SE [n = 40 for year].

### 3.2 Chlorophyll and carotenoid content

**Figure 1:**
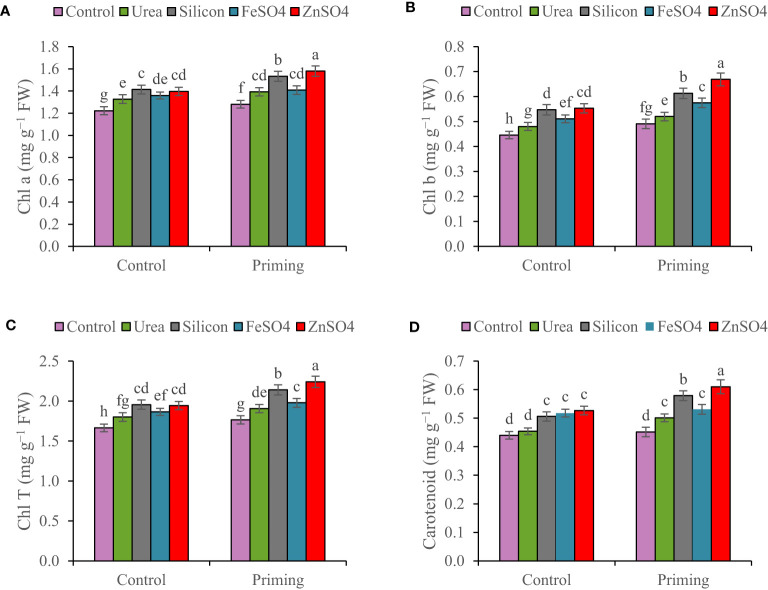
Mean comparison for interactions of seed priming × foliar application on **(A)** chlorophyll a (Chl a), **(B)** chlorophyll b (Chl b), **(C)** total chlorophyll (Chl T), and **(D)** carotenoid of wheat. The Duncan’s test indicates that columns labeled by the same letter are not significantly different at the *p* ≤ 0.05 level. Each mean is accompanied by a standard error (*n* = 8).

In the 2022–2023 growing season, Chl a, Chl b, Chl T, and carotenoid concentrations were 13.85%, 18%, 15.08%, and 14.58% higher than in the 2021–2022 growing season, respectively (**Table 3**). Leaf chlorophyll content for both primed and unprimed seeds decreased in order to ZnSO_4_ > silicon > FeSO_4_ > urea > control foliar application. Foliar application of ZnSO_4_, silicon, FeSO_4_, and urea at seed-primed treatment increased total chlorophyll content by 26.97%, 21.25%, 12.07%, and 7.99%, respectively, compared to the control. Furthermore, silicon, ZnSO_4_, FeSO_4_, and urea applied as foliar treatments at non-primed seed treatment increased Chl T by 17.48%, 16.83%, 12.08%, and 8.23% in comparison with the control (**Figures 1A–C**). Maximum carotenoid content (0.610 mg g^−1^ FW) was recorded with seed priming and ZnSO_4_ foliar application, which was significantly higher compared to other treatments (**Figure 1D**).

### 3.3 Proline concentration

**Figure 2:**
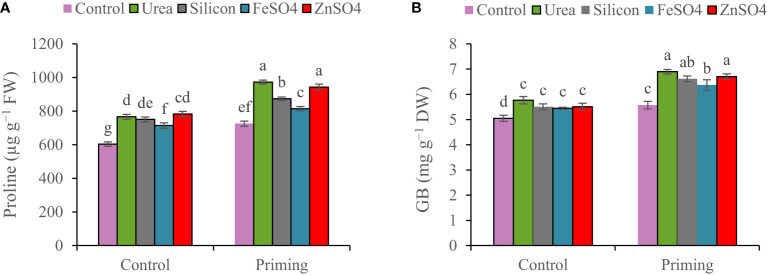
Mean comparison for interactions of seed priming × foliar application on concentrations of **(A)** proline and **(B)** glycine betaine (GB) in wheat. The Duncan’s test indicates that columns labeled by the same letter are not significantly different at the *p* ≤ 0.05 level. Each mean is accompanied by a standard error (*n* = 8).

Proline concentration was 6.65% higher in the first growing season than in the second growing season (**Table 3**). For all foliar applications, the highest proline concentration was obtained during primed seed treatment. Proline concentration ranged from 604.1 µg g^−1^ FW in non-primed seed and control foliar application to 942.7 µg g^−1^ FW in combination primed seed and urea foliar application. Seed priming recorded greater proline concentrations (972.8, 942.7, 873.4, 814.1, and 725.5 µg g^−1^ FW) at foliar application of urea, ZnSO_4_, silicon, and FeSO_4_ and no foliar application (**Figure 2A**).

### 3.4 GB concentration

A higher concentration of GB was observed in the 2021–2022 growing season (6.08 mg g^−1^ DW) than in the 2022–2023 growing season (5.86 mg g^−1^ DW). The highest GB concentration (6.71 mg g^−1^ DW) was observed at the co-application of seed priming and foliar spraying of urea. Foliar application of urea, ZnSO_4_, silicon, and FeSO_4_ compared to control at seed priming treatment elevated GB concentrations by 19.22%, 15.86%, 14.31%, and 10.06%, respectively (**Figure 2B**).

### 3.5 MDA concentration

**Figure 3:**
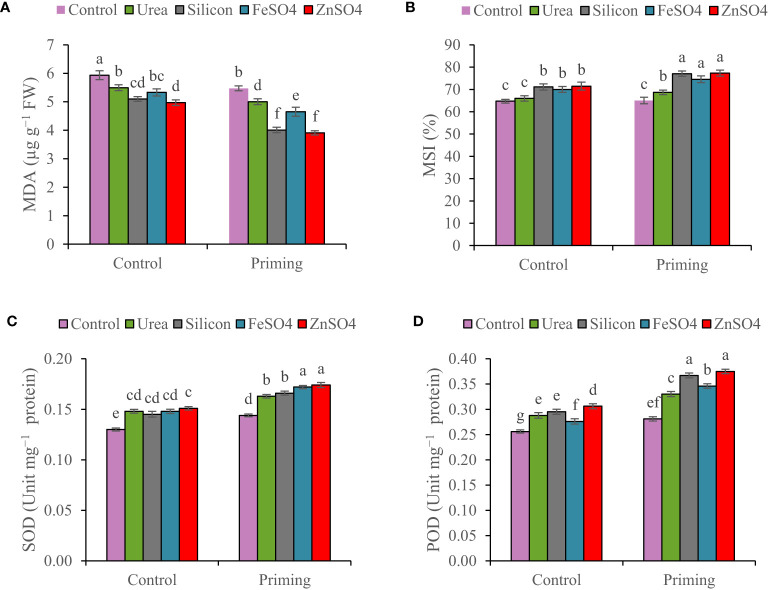
Mean comparison for interactions of seed priming × foliar application on **(A)** malondialdehyde concentration (MDA), **(B)** membrane stability index (MSI), **(C)** superoxide dismutase (SOD), and **(D)** peroxidase activity (POD) of wheat. The Duncan’s test indicates that columns labeled by the same letter are not significantly different at the *p* ≤ 0.05 level. Each mean is accompanied by a standard error (*n* = 8).

The concentration of MDA decreased by 7.15% in the second growing season compared to the first growing season (**Table 3**). The results of the study confirm that, as a result of foliar applications, the MDA concentration in wheat leaf was significantly smaller at primed and non-primed treatments. However, the outcomes of foliar application of ZnSO_4_ and silicon resulted in a greater reduction in MDA concentrations than other foliar applications. The highest MDA concentration (6.19 µg g^−1^ FW) was detected in the control foliar application and non-primed seed. Foliar application of ZnSO_4_, silicon, FeSO_4_, and urea compared to control at seed priming treatment reduced MDA concentrations by 30.61%, 28.80%, 17.40%, and 11.17%, respectively (**Figure 3A**).

### 3.6 MSI

The MSI increased from 68.5% in the 2021–2022 growing season to 72.7% in the 2022–2023 growing season (**Table 3**). MSI values ranged from 64.71% in non-primed seed and no foliar application to 77.321% in primed seed and silicon foliar application. Seed priming recorded significantly elevated MSI (77.32%, 77.07%, 74.55%, and 68.68%) at foliar application of ZnSO_4_, silicon, FeSO_4_, and urea, respectively (**Figure 3B**).

### 3.7 SOD activity

In both non-primed seed and seed primed treatments, ZnSO_4_, urea, silicon, and FeSO_4_ foliar applications significantly improved SOD activity. SOD activity values ranged from 0.13 Unit mg^−1^ protein in non-primed seed and no foliar application to 0.174 Unit mg^−1^ protein in primed seed and ZnSO_4_ foliar application. ZnSO_4_, FeSO_4_, silicon, and urea applications significantly increased SOD activity compared to no application in the seed priming treatment by 20.83%, 19.44%, 15.28%, and 13.19%. For non-primed seeds, there is no statistically significant difference in SOD activity between ZnSO_4_, silicon, FeSO_4_, and urea foliar applications (**Figure 3C**).

### 3.8 POD activity

Peroxidase activity in 2021–2022 was higher than the 2022–2023 growing season (**Table 3**). At both non-primed seed and seed primed treatments, ZnSO_4_, urea, silicon, and FeSO_4_ foliar applications significantly boosted POD activity. The highest POD activity (0.375 Unit mg^−1^ protein) was observed in seed priming in combination with ZnSO_4_ foliar application. This was statistically similar to the foliar application of silicon (0.367 Unit mg^−1^ protein) at seed priming treatment. In seed priming treatment, foliar application of ZnSO_4_, silicon, FeSO_4_, and urea significantly increased POD activity compared to no foliar application by 33.45%, 30.60%, 23.13%, and 17.44%, respectively (**Figure 3D**).

### 3.9 Yield and yield components

**Figure 4:**
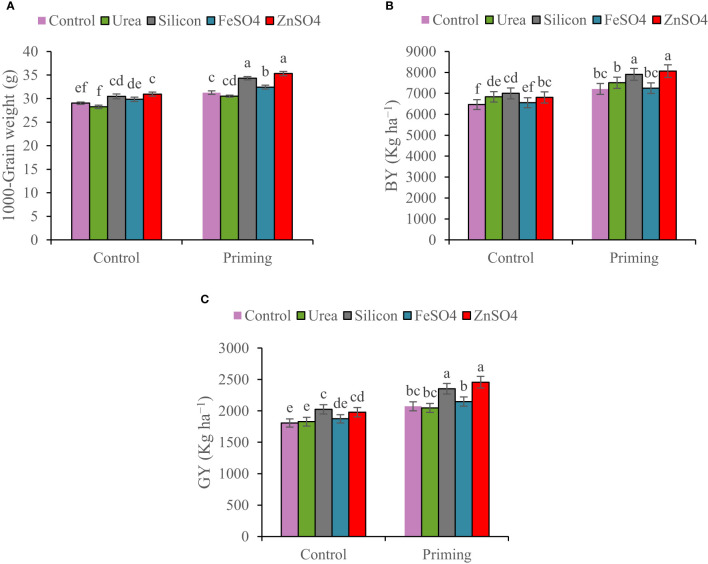
Mean comparison for interactions of seed priming × foliar application on **(A)** 1,000-grain weight, **(B)** biological yield (BY), and **(C)** grain yield (GY) of wheat. The Duncan’s test indicates that columns labeled by the same letter are not significantly different at the *p* ≤ 0.05 level. Each mean is accompanied by a standard error (*n* = 8).

In the 2022–2023 growing season, spike per m^2^, kernels per spike, 1000-grain weight, BY, and GY were 22.63%, 7.84%, 7.64%, 46.52%, and 45.50% higher than in the 2021–2022 growing season, respectively (**Table 4**). A seed priming treatment resulted in an overall improvement in yield components and yield. In comparison to the control, seed priming increased spikes per m^2^ and kernels per spike by 8.60% and 13.42%, respectively. A higher HI was found in the seed priming (29.2%) treatment compared to the control treatment (28.9%) (**Table 4**). Data in **Table 4** indicated that kernels per spike increased in the order control < ZnSO_4_ < silicon < FeSO_4_ < urea foliar applications. Foliar application of ZnSO_4_, silicon, and FeSO_4_ had a positive effect on 1,000-grain weight at both seed priming and control treatments, in contrast to foliar application of urea, which slightly decreased 1,000-grain weight. The maximal 1,000-grain weight on the whole foliar application was observed in ZnSO_4_ foliar application at both priming (35.30 g) and control (30.96 g) treatments (**Figure 4A**). For all foliar applications, the highest BY and GY were obtained at primed seed treatment. Biological yield ranged from 6,466 kg ha^−1^ in non-primed seed and control foliar application to 8,062 kg ha^−1^ in combination primed seed and ZnSO_4_ foliar application (**Figure 4B**). The highest GY (2,456 kg ha^−1^) was observed in seed priming in combination with ZnSO_4_ foliar application. This was statistically similar to the foliar application of silicon (2,353 kg ha^−1^) at seed priming treatment. Foliar application of ZnSO_4_ at seed primed and non-primed seed treatments increased GY by 18.51% and 9.60%, respectively. In addition, silicon foliar application at seed primed and non-primed seed treatments improved GY by 13.52% and 12.15%, respectively (**Figure 4C**). According to the results of the pot experiments, seed primed attained enlarged aboveground DM, grain weight, root DM, root volume, and root length by 9.56%, 10.56%, 9.64%, 8.95%, and 4.37%, respectively (**Table 4**).

**Table 4:** The mean comparisons of spike per m^2^, kernels per spike, biological yield (BY), grain yield (GY), harvest index (HI), and grain Zn, Fe, and protein content of wheat are influenced by seed priming (SP) and foliar applications (FA).

Mean comparisons		Spike per m^2^	Kernels per spike		HI (%)		Zn		Fe			Protein (%)	
							(mg kg^−1^ DW)						
Year													
**2021–2022**		402^b^ ± 5.35	15.3^b^ ± 0.20		28.9^a^ ± 0.24		40.2^a^ ± 0.76		53.3^a^ ± 0.73			11.1^a^ ± 0.08	
**2022–2023**		493^a^ ± 6.84	16.5^a^ ± 0.23		28.6^a^ ± 0.23		41.2^a^ ± 0.87		53.8^a^ ± 0.89			11.6^a^ ± 0.10	
**Priming**													
**Control**		430 ^a^ ± 6.87	14.9^b^ ± 0.18		28.3^b^ ± 0.20		39.4^b^ ± 0.68		52.1^b^ ± 0.69			11.5^b^ ± 0.09	
**Priming**		467^a^ ± 7.25	16.9^a^ ± 0.18		29.2^a^ ± 0.24		42.0^a^ ± 0.88		55.0^a^ ± 0.86			11.8^a^ ± 0.10	
Foliar application													
**Control**		448^a^ ± 12.39	15.0^d^ ± 0.34		28.3^b^ ± 0.29		38.2^b^ ± 0.64		51.1^b^ ± 0.82			11.1^c^ ± 0.15	
**Urea**		460^a^ ± 13.89	15.5^cd^ ± 0.28		27.1^c^ ± 0.27		38.0^b^ ± 0.46		51.6^b^ ± 0.55			12.5^a^ ± 0.14	
**Silicon**		456^a^ ± 12.28	16.3^ab^ ± 0.37		29.4^a^ ± 0.26		38.7^b^ ± 0.60		51.7^b^ ± 0.95			11.4^bc^ ± 0.08	
**FeSO_4_**		437^a^ ± 10.96	16.0^bc^ ± 0.32		29.2^a^ ± 0.30		39.2^b^ ± 0.90		61.1^a^ ± 0.83			11.6^b^ ± 0.09	
**ZnSO_4_**		442^a^ ± 11.81	16.7^a^ ± 0.38		29.8^a^ ± 0.36		49.4^a^ ± 0.95		52.1^b^ ± 0.92			11.6^b^ ± 0.13	
**Year**		1,000-grain weight (g)			BY				GY				
					(kg ha^−1^)								
**2021–2022**		30.1^b^ ± 0.32			5,808^b^ ± 63.0				1,677^b^ ± 30.09				
**2022–2023**		32.4^a^ ± 0.40			8,510 ^a^ ± 95.4				2,440^a^ ± 41.50				
**The mean comparisons of aboveground dry mass, grain weight, root dry mass, root length, and root volume wheat in the pot experiment.**												
	Dry mass weight			Grain weight		Root dry mass		Root length (cm)			Root volume (cm3 pot^−1^)		
	(g pot^−1^)												
**Control**	13.6^b^ ± 0.33			5.49^b^ ± 0.21		1.66^b^ ± 0.04		5.03^b^ ± 0.11			84.7^b^ ± 1.1	
**Priming**	14.9^a^ ± 0.48			6.07^a^ ± 0.12		1.82^a^ ± 0.06		5.48^a^ ± 0.16			88.4^a^ ± 0.92	

As determined by Duncan’s test, no significant difference at p ≤ 0.05 exists between values in a column containing the same letter within a group. Data are the mean ± SE [n = 40 for year; n = 40 for seed priming; n = 16 for foliar application; n = 4 for seed priming in the pot experiment].

### 3.10 Grain Fe and Zn content

Grain Fe (55.0 mg kg^−1^ DW) and Zn (42.0 mg kg^−1^ DW) content in seed priming treatment was significantly higher than control. Maximum grain zinc content (49.4 mg kg^−1^ DW) was achieved in ZnSO_4_ foliar spray. In comparison to the control, foliar applications of ZnSO_4_ enhanced grain Zn content by 29.32%. Only FeSO_4_ foliar application significantly increased grain Fe content compared to no foliar application. Grain Fe content ranged from 51.1 mg kg^−1^ DW for no foliar application to 61.1 mg kg^−1^ DW for FeSO_4_ foliar application (**Table 4**).

### 3.11 Grain protein content

Because of seed priming, grain protein content increased from 11.5% to 11.8% in comparison with control. Overall, urea, ZnSO_4_, FeSO_4_, and silicon applied foliarly improved grain protein content as compared with control. However, silicon foliar applications are statistically similar to no foliar application in grain protein content. The protein content of grains ranged from 11.1% for no foliar application to 12.5% for urea foliar application (**Table 4**).

### 3.12 Correlation among traits

According to **Table 5**, there is a significant positive association between GY and all physiological traits except MDA concentration (*r* = −0.62, *p* ≤ 0.01). Furthermore, there was a positive association among GY with BY (*r* = 0.97, *p* ≤ 0.01), spike per m^2^ (*r* = 0.75, *p* ≤ 0.01), kernels per spike (*r* = 0.69, *p* ≤ 0.01), 1,000-grain weight (*r* = 0.61, *p* ≤ 0.01), and Zn content in grains (*r* = 0.35, *p* ≤ 0.01). A positive correlation was found between BY, spike per m^2^, kernels per spike, 1,000-grain weight, MSI, SOD and POD activity, Chl T, carotenoid, proline concentration, and grain Zn content. MSI was positively associated with Chl T (*r* = 0.75, *p* ≤ 0.01), carotenoid (*r* = 0.76, *p* ≤ 0.01), proline (*r* = 0.44, *p* ≤ 0.01), GB (*r* = 0.49, *p* ≤ 0.01), SOD activity (*r* = 0.63, *p* ≤ 0.01), POD activity (*r* = 0.64, *p* ≤ 0.01), grain Zn content (*r* = 0.47, *p* ≤ 0.01), and grain Fe content (*r* = 0.35, *p* ≤ 0.05), while MSI showed a strong negative relation with MDA content (*r* = −0.75, *p* ≤ 0.01). The MSI is positively related to all traits except grain protein content while there is a significant negative relation between MDA and all traits investigated excluding grain protein content (no significant correlation between MDA with grain protein content). There was no significant relationship between Zn, Fe, and protein content of grains. Grain protein content exclusively had a significant association with proline and GB concentrations, and SOD and POD activity (**Table 5**).

**Table 5:** Correlation coefficients of grain yield (GY), biological yield (BY), harvest index (HI), spike per m2 (SP), kernels per spike (KS), 1,000-grain weight (GW), total chlorophyll (Chl T) and carotenoid (Car) content, membrane stability index (MSI), proline, glycine betaine (GB), and malondialdehyde (MDA) concentration, superoxide dismutase (SOD), peroxidase activity (POD), grain Zn content (Zn), grain Fe content (Fe), and grain protein content (GPC) of wheat are affected by priming levels and foliar applications.

	GY	BY	HI	SP	KS	GW	Chl T	Car	MSI	Proline	GB	MDA	SOD	POD	Zn	Fe	GPC
**GY**	1																
**BY**	0.97^**^	1															
**HI**	0.30**	0.06^ns^	1														
**SP**	0.75^**^	0.80^**^	0.05^ns^	1													
**KS**	0.69^**^	0.62^**^	0.39^**^	0.49^**^	1												
**GW**	0.61^**^	0.49^**^	0.57^**^	0.30^**^	0.75^**^	1											
**Chl T**	0.85^**^	0.78^**^	0.43^**^	0.53^**^	0.75^**^	0.75^**^	1										
**CAR**	0.77^**^	0.69^**^	0.44^**^	0.47^**^	0.70^**^	0.73^**^	0.92^**^	1									
**MSI**	0.61^**^	0.55^**^	0.32^**^	0.37^**^	0.67^**^	0.63^**^	0.75^**^	0.76^**^	1								
**Proline**	0.26^*^	0.23^*^	0.22^*^	0.17^ns^	0.58^**^	0.54^**^	0.51^**^	0.46^**^	0.44^**^	1							
**GB**	0.23^*^	0.21^ns^	0.28^*^	0.18^ns^	0.52^**^	0.52^**^	0.44^**^	0.44^**^	0.49^**^	0.83^**^	1						
**MDA**	−0.62^**^	−0.52^**^	−0.50^**^	−0.35^**^	−0.75^**^	−0.83^**^	−0.82^**^	−0.81^**^	−0.75^**^	−0.61^**^	−0.63^**^	1					
**SOD**	0.30^**^	0.22^*^	0.41^**^	0.17^*^	0.63^**^	0.61^**^	0.55^**^	0.54^**^	0.63^**^	0.74^**^	0.80^**^	−0.73^**^	1				
**POD**	0.30^**^	0.24^*^	0.44^**^	0.10^ns^	0.64^**^	0.71^**^	0.59^**^	0.59^**^	0.64^**^	0.77^**^	0.80^**^	−0.79^**^	0.87^**^	1			
**Zn**	0.35^**^	0.25^*^	0.40^**^	0.08^ns^	0.46^*^	0.54^**^	0.6^**^	0.57^**^	0.47^**^	0.45^**^	0.38^**^	−0.48^**^	0.46^**^	0.52^**^	1		
**Fe**	0.10^ns^	0.05^ns^	0.25^*^	0.11^ns^	0.23	0.18^ns^	0.14^ns^	0.27^*^	0.35^**^	0.10^ns^	0.33^**^	−0.24^*^	0.46^**^	0.25^*^	0.04^ns^		
**GPC**	−0.10^ns^	−0.03^ns^	−0.19^ns^	0.02^ns^	0.07^ns^	−0.07^ns^	0.03^ns^	−0.01^ns^	−0.01^ns^	0.52^**^	0.49^**^	−0.10^ns^	0.40^**^	0.29^**^	0.04^ns^	0.16^ns^	1

ns, non-signiﬁcant; *p ≤ 0.05; **p ≤ 0.01.

## 4 Discussion

Uneven precipitation patterns in Mediterranean areas during rainfed wheat growing lead to soil moisture shortages (Moradi et al., 2022). In such situations, integrated agronomic crop management practices are required to alleviate drought stress’s negative impacts (Melash et al., 2019). Seed priming (Liu et al., 2015; Rai-Kalal and Jajoo, 2021) and foliar application of nutrients and different compounds (Bukhari et al., 2021; Malik et al., 2021; Ghani et al., 2022) are two drought amelioration techniques. The combination of these practices can perform a key role in elevating plant tolerance to drought stress and improving GY and quality.

N, Fe, and Zn are essential nutrients needed for growth of plant and are essential components of a variety of critical physiological processes (Zain et al., 2015; Nehe et al., 2020; Sattar et al., 2021). Silicon is a nutrient that is currently receiving heightened interest from the scientific community, most notably in its application to drought resistance (Ayed et al., 2021; Bukhari et al., 2021; Sattar et al., 2021). Utilization of these elements through nutritional seed priming is considered a cost-efficient approach since only a small amount of nutrients are utilized to prime and ensure nutrient availability for germinating seeds (Farooq et al., 2021). In the late growth stages of rainfed wheat, insufficient soil moisture and limited root activity result in a reduction in nutrient uptake (Moradi et al., 2022). In the present study, the crop was exposed to a continuous increase in ambient temperature with a lack of atmospheric moisture and a decrease in soil moisture storage, especially during grain filling due to lack of rainfall. As a consequence, foliar application of nutrients during the final stages of growth, when soil nutrient application is impossible due to moisture limitations, leads to rapid absorption by the leaves (Aziz et al., 2019; Melash et al., 2019). In this study, it was observed that seed priming and foliar spraying of ZnSO_4_, silicon, FeSO_4_, and urea alleviated adverse drought effects and improved plant physiological properties (**Figures 1A–D**, **2A, B**, **3A–D**).

Chlorophyll and carotenoids, which are pigments essential for photosynthesis, also play a crucial role (Kiran et al., 2021; Zhang et al., 2023). Water shortages exacerbate nutrients such as N, Zn, and Fe deficiency that reduce leaf pigment content due to a decrease in pigment synthesis, a reduction in the enzyme activity engaged in photosynthetic pigment synthesis, and a rise in photosynthetic pigment degradation (Kiran et al., 2021; Moradi et al., 2022; Zhang et al., 2023). Chlorophyll and carotenoid content are indicators of plant resistance to environmental stresses because it is directly linked with photosynthesis (Ayed et al., 2021). Nevertheless, our results indicated that overall chlorophyll and carotenoid content in primed seed was greater than in non-primed seed; foliar application (especially ZnSO_4_ and silicon) increased photosynthetic pigments in both primed and unprimed seed treatments; however, foliage spraying had the greatest effect in primed seeds (**Figures 1A–D**). The increase in photosynthetic pigment contents due to priming and foliar application treatments enhances nutrient availability and water conservation (Hussain et al., 2019; Farooq et al., 2021). Numerous studies indicated that seed priming and foliar spraying of N, Fe, Zn, and silicon mitigated the injurious impacts of drought stress and enhanced leaf carotenoid and chlorophyll content (Hussain et al., 2019; Melash et al., 2019; Ayed et al., 2021; Farooq et al., 2021; Sattar et al., 2021). In the same way, our observations are also supported by Khan et al. (2022) and Anwar et al. (2021), who reported that seed priming and foliar application improved photosynthetic pigments. The higher content of photosynthetic pigments as a result of foliar spray and priming of nutrients can be associated with the presence of these elements in the structure of pigments (such as N in chlorophyll structure) and their effect on reducing negative effects of drought stress and ultimately preserving pigments. Increased leaf chlorophyll and carotenoid concentrations were detected under seed priming and foliar application, which not only improved photosynthesis but likewise enhanced crop growth, GY, and grain quality.

Under drought stress, plants maintain potential water balance and cellular metabolism by synthesizing and collecting compatible osmolytes such as GB and proline. Owing to osmolytes’ functions of regulating osmotic pressure, maintaining turgor pressure, and regulating cell volume, metabolic activity is preserved under low water potential. Plants can also utilize GB and proline as nitrogen and carbon sources under extreme conditions (Tian et al., 2017; Bukhari et al., 2021). In the current research, seed priming and foliar application improved proline and GB concentrations. Foliar spraying of nutrients (mainly N and Zn) enhanced these compatible osmolytes in primed and non-primed seed treatment; however, proline and GB concentration improvement in primed seed was highest (**Figures 2A, B**). Foliar application of nutrients and seed priming preserve the plant and enhance growth by stimulating the synthesis of osmolytes and maintaining osmotic potential when water is scarce (Malik et al., 2021; Sattar et al., 2021; Saha et al., 2022). Various enzymatic antioxidants, such as SOD and POD, as well as non-enzymatic antioxidants, participate in ROS detoxification (Khan et al., 2023). Proline and GB are non-enzymatic antioxidants that protect the plant from negative effects by stabilizing reactive oxygen species (ROS; Malik et al., 2021; Malko et al., 2022). Several factors may have contributed to this elevation in GB and proline accumulation, including those attributed to the presence of these components in the structure of these osmolytes, as their participation in the structure of many enzymes engaged in the synthesis of these osmolytes as cofactors and the expression of genes encoding key enzymes involved in the synthesis of proline and GB have increased (Umair Hassan et al., 2020; Saha et al., 2022). Previous research demonstrated that foliar nutrients and seed priming raised proline and GB accumulation under drought stress (Bukhari et al., 2021; Malik et al., 2021; Singhal et al., 2021; Ghani et al., 2022).

Mineral nutrition acts as a key function in alleviating stress caused by drought in plants. Plants have several processes for dealing with drought damage, including enzymatic (for example POD) and antioxidants that are not enzyme-based (for example, proline and GB as free amino acids) (Malik et al., 2021; Malko et al., 2022). Seed priming and foliar application of nutrients reduce ROS damage via elevating enzymatic and non-enzymatic antioxidant contents and strengthening drought resistance in plants (Farooq et al., 2021; Malik et al., 2021; Sattar et al., 2021). In plants, antioxidants play an essential role in enhancing plant tolerance to stress in response to abiotic stresses (Khan et al., 2023). According to our results, seed priming and foliar application positively affected antioxidant enzymes (POD and SOD) activity. Seed priming together with foliar application of silicon, ZnSO_4_, FeSO_4_, and urea compared to foliar application of these compounds in unprimed seed increased POD activity by 24.40%, 22.55%, 25.36%, and 14.58%, respectively. Likewise, POD activity in no foliar application and primed seed treatment was 9.77% higher than in no foliar application and non-primed seed (**Figure 3D**). This study demonstrated that seed priming and foliar application of nutrients and elevated nutrient availability have a positive effect on POD and SOD activity, because it is due to the presence of these nutrients in the enzyme structure as well as many antioxidants contain this compound as cofactors (Karim et al., 2012; Rehman et al., 2018; Umair Hassan et al., 2020; Kiran et al., 2021; Saha et al., 2022). In previous research, it has been displayed that seed priming and foliar application of these components enhance the enzyme-based antioxidant defenses of plants (Kiran et al., 2021; Lv et al., 2021; Malik et al., 2021; Sattar et al., 2021; Singhal et al., 2021; Khan et al., 2022; Raza et al., 2023).

Stress induced by water and nutrient deficiency results in oxidative stress in plants, causing membrane lipid peroxidation. Tissue MDA amounts represent membrane lipid peroxidation, which eventually damages integrity of membranes (Chavoushi et al., 2020; Farooq et al., 2021). The MSI is widely recognized as a physiological marker and stress tolerance evaluation tool. Abiotic tolerance in plants relies heavily on maintaining membrane integrity and stability since membranes are the main targets of environmental stresses (Malik et al., 2021). The contents of the cell leak out when the membrane of cell is disrupted, reducing cell membrane development and elevating electrolyte leakage (Malik et al., 2021; Singhal et al., 2021). In this study, seed priming and foliar spray of nutrients, and a co-application of these techniques, reduced MDA concentration and improved MSI (**Figures 3A, B**). The results of this experiment suggest that seed priming enhances root growth (**Table 4**) and increases access to water and nutrients. Nutrient supply via foliar spray and seed priming modulated ROS adversity by reducing the rate of lipid peroxidation (**Figure 3A**). As a result of regulating antioxidant mechanisms (**Figures 3C, D**) and maintaining lipid biological membranes (**Figure 3B**), it was evident that enzymatic activity (SOD and POD activity) had a strong positive correlation with non-enzymatic (carotenoid, proline, and GB concentration) antioxidants and MSI, and there was a significant negative correlation between MDA concentration with SOD and POD activity and carotenoid, proline, and GB concentration (**Table 5**). Our result is supported by previous studies that indicated that nutrient supply by foliar spray and seed priming stabilizes the membranes and reduces MDA concentration, which consequently mitigates oxidative stress and lowers membrane injury (Hussain et al., 2019; Chavoushi et al., 2020; Ayed et al., 2021; Farooq et al., 2021; Malik et al., 2021; Raza et al., 2023).

The growth, yield components, and GY are strongly related and the efficiency of one influences the other. Furthermore, a significant boost in growth and yield components improves the GY (Malko et al., 2022). According to this study, the seed priming had a positive influence on the kernels per spike, 1,000-grain weight, BY, GY, HI, and root growth (**Table 4**; **Figure 4**). Recently, numerous researchers (Rehman et al., 2018; Hussain et al., 2019; Ayed et al., 2021; Raza et al., 2023) reported that seed priming increased yield components and yield that supported our results. Enhanced tolerance in plants grown from a primed seed resulted in improved root development and, consequently, assisted in improved water and nutrition uptake under water scarcity conditions (Farooq et al., 2021). During the reproductive stage, water shortage, reduction in root activity, and nutrient deficiency in rainfed wheat affect grain development, kernels per spike, and grain weight, eventually reducing the yield and quality of final products (Saha et al., 2022). The present research found that foliar spray of ZnSO_4_, silicon, and FeSO_4_ during the terminal stage significantly increased kernels per spike, 1000-grain weight, BY, and GY in comparison with the control (**Table 4** and **Figure 4**). Furthermore, urea foliar application significantly enhanced BY compared with the control (**Figure 4**). In this study, it seems that urea foliar spray, despite staying green (maintaining photosynthetic capacity and improving current photosynthesis) and increasing BY, has delayed leaf senescence and remobilization of dry matter to grain; ultimately, it had no significant effect on GY (the lowest HI obtained in urea foliar spray treatment). In the past, researchers have consistently published that foliar application of nutrients significantly improved yield components and GY (Karim et al., 2012; Zain et al., 2015; Sultana et al., 2018; Kiran et al., 2021). Analyses of correlation clearly confirmed that GY was closely associated with physiological characteristics (**Table 5**). This study describes that seed priming and foliar application of nutrients improved drought resistance through the increase in compatible osmolytes (GB and proline) and carotenoid concentrations (**Figure 1D**; **Figure 2**) and antioxidant enzyme (POD and SOD) activity (**Figure 3B**), decrease in MDA concentration (**Figure 3A**), and maintenance of cell membrane integrity (**Figure 3B**); prevented chlorophyll degradation (**Figures 1A–C**); and eventually enhanced GY (**Figure 4C**). Our results indicated that chlorophyll content, MSI (**Table 3**), spike per m^2^, kernels per spike, 1,000-grain weight, and BY (**Table 4**) in plants during 2022–2023 were higher than those during the 2021–2022 growing season (**Table 5**; **Figure 2**), which might be associated with more precipitation in the second growing season (**Table 2**).

In this study, seed priming and nutrient foliar application influenced grain quality. We found that seed priming significantly increased grain Fe, Zn, and protein content (**Table 4**). An adequate nutrient source is extremely vital in seed germination and the initial stages of development; nutritional seed priming improves root growth, nutrient uptake, and plant growth (Farooq et al., 2021; Malko et al., 2022; Saha et al., 2022). Results of our pot experiment indicate that root growth is significantly improved by seed priming (**Table 4**). These findings suggest that the mechanism of improved grain quality by seed priming practice is based on the improvement in boosted root growth and development, enhanced nutrient absorption, and translocation of nutritional elements from root to shoot and grain, and ultimately led to improved grain quality (grain Zn, Fe, and protein content). This finding was in line with previous investigations suggesting that seed priming improves grain quality (Rehman et al., 2018; Anwar et al., 2021; Singhal et al., 2021). The application of nutrients in the terminal stage of rainfed wheat due to the high efficiency of foliar spraying is rapidly absorbed by plants. This permits nutrients to be transported directly to points with high metabolic demand (grains). Earlier studies showed that foliar nutrient application could boost grain quality (Melash et al., 2019; Sultana et al., 2018; Kiran et al., 2021; Kiran et al., 2021). In the current study, foliar nutrient application also improved grain quality. Foliar application of ZnSO_4_, FeSO_4_, and urea drastically boosted grain Zn, Fe, and protein content, respectively (**Table 4**). Thus, foliar nutrient delivery at the terminal stage is a high-efficiency method for biofortification in rainfed wheat cultivation. The farmer community can use seed priming and nutrient foliar application at the terminal stage to mitigate the adverse effects of drought stress and improve growth attributes, grain yield, and quality of rainfed wheat.

## 5 Conclusion

Results from this study have clearly demonstrated that the combination of seed priming [by urea (20 g L^−1^) + FeSO_4_.7H_2_O (50 ppm) + ZnSO_4_.7H_2_O (50 ppm) + silicon (20 mg L^−1^) solution] and foliar application of these compounds [urea (4%), silicon (4 mM), FeSO_4_.7H_2_O (0.6%), and ZnSO_4_.7H_2_O (0.4%)] improved physiological and yield characteristics. Foliar application of ZnSO_4_, FeSO_4_, and urea drastically affected grain Zn, Fe, and protein content, respectively. To improve drought resistance, GY, and quality of rainfed wheat, seed priming and foliar spraying are recommended as crop management practices. Future investigations are necessary to assess the influence of seed priming and foliar spray of these compounds at various growth stages on rainfed wheat in various cultivars and soils.

## Data availability statement

The original contributions presented in the study are included in the article/supplementary material. Further inquiries can be directed to the corresponding author.

## Author contributions

LM: Formal Analysis, Project administration, Software, Validation, Writing – original draft. AS: Funding acquisition, Investigation, Methodology, Project administration, Resources, Writing – review & editing.
